# Silicea

**Published:** 1898-02

**Authors:** 


### HOMŒOPATHIC PHYSICIAN,

A MONTHLY JOURNAL OF

HOMŒOPATHIC MATERIA MEDICA AND CLINICAL MEDICINE.

"If our school ever gives up the strict inductive method of Hahnemann, we are lost, and deserve only to be mentioned as a caricature in the history of medicine."—CONSTANTINE HERING.

Vol. XVIII. FEBRUARY, 1898. No. 2.

### EDITORIAL.

SILICEA.—The lively conscientiousness and compunction of conscience about trifles is a reminder of Carbo-vegetabilis, which has a great deal of anxiety, as if he had committed a crime, causing trembling and weeping.

Obstinacy of children under Silicea is matched by the same defect of character under Calcarea-carbonica.

The headaches of Silicea begin about noon and continue all the afternoon.

The headaches of Silicea are relieved by wrapping up the head warmly. This is a great characteristic of Silicea, and should always be remembered when we have cases of headache to attend.

A case of headache relieved by warm wraps around the head is reported in Skinner's *Organon*, Volume I, page 264, Case 3. Silicea was given and brought about a complete cure.

Silicea is indicated in the open fontanelles of young babies. Calcarea also is useful. Under Silicea the head is too large, while the body is emaciated, and the face pale and waxy. Calcarea has pale, bloated face.

Silicea has, with the above-mentioned condition of head and body, hot, swollen abdomen, and very fetid stools. Calcarea has hard swelling of the abdomen, like a saucer turned bottom upward, to use the figure of the late Dr. H. N. Guernsey, and there are also sour stools.

Silicea has sour-smelling perspiration on the head in children, and so has Calcarea-carbonica. Under Calcarea the perspiration begins as soon as the child is asleep. Under Silicea this perspiration occurs in the morning hours of the night. Thus the difference between Calcarea and Silicea is that the perspiration occurs on the head during sleep, before midnight, under Calcarea, while the perspiration of Silicea occurs on the head during sleep after midnight, and toward morning.

Lachrymation in the open air occurs under both Silicea and Phosphorus. Agglutination of the eyes in the morning on waking occurs under Silicea, Phosphorus, and Pulsatilla.

Silicea has swelling of the tear gland of the left eye, and Bromine has same thing in the right eye.

Swelling of the lachrymal duct from occlusion indicates Silicea, especially if it be in the left eye. Bromine has the same trouble in the right eye, and under Pulsatilla the accumulation becomes pus-like.

The editor, when only a student of medicine, had a case of occlusion of the lachrymal duct occurring in his own mother. There was fistula, and the tears flowing down the duct and out through the fistula swelled up the tissues in the inner corner of the left eye considerably. This continued for several years, until finally pus was secreted which swelled up the tumor in the left inner canthus until it was the size of a walnut; then ulceration of the external skin at the highest point occurred, a fistula was formed, the pus discharged externally, and the tumor collapsed and remained so for several hours, when it would begin to rise again. Eminent physicians tried to cure it, and failed. Surgeons proposed operation, which was refused, and finally the writer of this article studied the case, thought that Silicea was indicated, gave it in the two-hundredth potency, and the tumor discharged and collapsed, and never rose again for twenty years. This certainly is a remarkable result to attain in a case that seemed to be suitable only for surgical interference. It is an encouragement to any student to persevere in the good cause.

Under Silicea there is confusion of vision with running together of the letters when looking at a printed page. This is similar to Natrum-muriaticum. The editor has restored sight and obviated the need of wearing glasses in a young girl of less than fourteen years of age who had this symptom whenever she attempted to study her lessons. Silicea was given, and the girl was able to do without glasses. It was the opinion of Dr. Lippe that the day would come when indicated remedies would be given for all those defects of vision which at present are treated with glasses. His opinion was shared also by the late Dr. H. N. Guernsey. Dr. Lippe especially objected to the placing of glasses upon young people. As is well known to the readers of this journal, it is no uncommon thing nowadays to see children of four years of age wearing glasses. But Dr. Lippe, in the prime of his professional activity, never witnessed such a thing. It has come about only within the last ten years, and Dr. Lippe has been dead ten years last January.

Silicea has aversion to light, especially daylight, as it produces much dazzling. Many remedies have this symptom, and it is, therefore, of not much value in selecting Silicea unless you have more characteristic symptoms of Silicea to correspond.

As a contrast to Silicea, Calcarea has aversion to candle or lamp light, because it is painful to the eyes.

Silicea has sudden attacks of blindness, with far-sightedness.

Silicea has stoppage of the ears, which open at times with a loud report. Calcarea has cracking in the ears while eating.

The loud, pistol-like report in the ears is a characteristic of Silicea.

Silicea has hard swelling of the parotid gland. Under Belladonna this swelling is highly inflammatory. Under Calcarea also it is highly inflammatory. It is the same with Rhus-toxicodendron, preferably on the left side. From these remarks it will appear that Belladonna, Calcarea-carbonica, Rhus-toxicodendron, and Silicea are of the highest value in the treatment of mumps.

Silicea and Calcarea both have painful dryness of the nose.

Silicea has ulcers high up in the nose with great sensitiveness to contact.

Kali-bichromicum has ulcers on septum of nose.

Thuja has ulcer covered over with a scab, which finally falls off, leaving a red surface.

Silicea has stoppage of nose. It also has acrid corroding discharge.

Nux-vomica has stoppage of the nose at night. If there be a discharge it is not acrid.

Nitric-acid has stoppage of the nose, and hot water runs out of it at the same time.

Thuja is the principal remedy for scabs in the nose.

Calcarea has ulcers in the nose, with thick, heavy discharge.

Silicea has sneezing in the morning, with much watery discharge from the nose.

Silicea has long-continued stoppage of the nose from hardened mucus.

Lycopodium has this obstruction, and it is located at the root of the nose.

Silicea and Calcarea both have ulcerated corners of the mouth.

This ulceration of the corners of the mouth is very characteristic of Nitric-acid.

